# Perpetuating ability to live life as usual: a grounded theory study of persons living with age-related macular degeneration

**DOI:** 10.1186/s12877-024-04689-9

**Published:** 2024-01-22

**Authors:** J. Källstrand, E-C. Lindgren, I. M. Carlsson

**Affiliations:** School of Health and Welfare, Halmstad University, Halmstad, Sweden

**Keywords:** Age-related macular degeneration, Grounded theory, Older adults, Visual impairment

## Abstract

**Background:**

Age-Related Macular Degeneration (AMD) is an eye disease associated with age that causes progressive and irreversible loss of central vision, while the peripheral visual ability remains. The occurrence of and especially late AMD is estimated to increase extensively to 2040 among persons aged ≥ 65 in Scandinavia, due to an increasing aging population.

**Objectives:**

The present study explored what it means to live with AMD through the eyes of those living with the condition.

**Methods:**

This is an explorative interview study. People who were ≥ 65 years old, living in their own homes, and diagnosed with advanced dry AMD in one or both eyes, causing a visual acuity of no more than 0.3 or worse in the best eye, were invited to participate in the study. The method chosen was the constructivist grounded theory, where reality is seen as fundamentally social and processual and a way of accessing the participants’ experiences, thoughts, and feelings.

**Results:**

In total, 12 interviews were conducted. Living with dry AMD confronted different problems and challenges. The substantive theory, Perpetuating ability to live life as usual, is characterised by a desire to continue life as usual, which requires an acceptance of the disease’s progress, self-acceptance of the new me, and an acceptance that the new life needs to be lived a little more carefully. Moreover, the participants used three strategies to resolve their main concern by maintaining an everyday life 1) Navigating the new normal, 2) Trusting own ability, and 3) Interdepending.

**Conclusion:**

Maintaining an everyday life is the primary concern among people with AMD. In supporting self-care, gaining information about the subjective experience to support their everyday living is of the utmost importance. This grounded theory captures valuable knowledge of how the older adults resolved their main concern “you got to keep on” despite their affected vision by “facing the fact” live life as usual since since life goes on. Our study also gives rise both to implications for research and practice in order to strengthen older people with AMD facing their future challenges.

**Trial registration:**

The Swedish Ethical Review Authority (EPN 2021/02877).

## Background

The global goal to promote well-being for all people of all ages is challenging due to an increasingly aging population, which is estimated to increase from 20.8% in 2021 to 31.3% in 2100 [1]. Today, aging is associated with maintaining independence [2, 3]. However, age is also associated with increasing disability, loss of independence and functional impairments, such as loss of vision [4, 5]. The third most common cause of impaired vision and legal blindness worldwide among people 60 years and older is age-related macular degeneration (AMD), which is progressive, and causing irreversible central vision loss [6, 7]. In a Swedish population-based study of 70-year-olds, as many as 4,7% reported having AMD [8], In Scandinavia though, the prevalence of early AMD was 3.5% for those aged 55–59 years and 17.6% for those aged 85 years and older, and because of advanced AMD, 0.1% and 9.8%, respectively [9]. However, as the aging population is estimated to increase, both the incidence and prevalence gets higher.

Because of the disease, almost every aspect of a person’s daily living is affected, especially visually demanding activities, which creates various challenges depending on the severity of the disease [10]. As AMD worsens, there is a risk of dependence in daily life, i.e., needing assistance [11]. Consequently, with respect to the challenges that visual impairment imposes on independent living, quality of life is affected by the decrease in visual acuity and the progress of AMD [6]. Even though the deterioration is relatively mild, there is a significant relationship between visual impairment and a reduced perceived vision-related quality of life [12]. Furthermore, older adults having problems with functioning caused by visual impairment are more likely to experience depression and anxiety [13].

With restrictions in daily activities, AMD also affects the person’s physical functioning with impaired mobility and fear of falling [14–17]. Studies have found that low daily physical activity levels increase the risk of functional decline, disability, and mortality [18]. Thus, persons with AMD are notably associated with falls and other injuries, such as bone fractures [19–21]. Considerable evidence now exists that habitually physical activity is essential for preserving physical function and mobility and preventing injuries and falls common to older people [18, 22, 23]. Research has also indicated that regular exercise may prevent AMD or delay its progression due to the association between physical activity and increased oxidative resistance [16].

### Context

This study is the Swedish part of a cross-border collaboration between Sweden and Denmark called “Can you see the future.” The project took place within a community in the southwest of Sweden with about 100 000 inhabitants [24]. It was designed to achieve a sustainable community that obtains health and well-being despite vision loss caused by advanced dry AMD. Therefore, exploring how people with advanced dry AMD perceive everyday life is essential to this aim, in accordance with the global goal to promote well-being for all people regardless of age due to the increasingly aging worldwide population.

## Methods

This study aims to develop a grounded theory of what it means to live with AMD through the eyes of those living with the disease. The design of this study was explorative. A constructivist-based grounded theory (CGT) was adopted, where reality is seen as fundamentally social and processual and a way of accessing the participants’ experiences, thoughts, and feelings [25]. This is an appropriate methodological choice when there is little pre-existing theory on a specific process, existing theories do not adequately capture the complexity of the process, and previous research is limited [25]. CGG can enhance the literature by offering new theoretical insights and explanations, producing rich and diverse data focusing on the participants experiencing dry AMD experiences. CGT was also selected as congruent with our philosophical perspective. Constructivism is a research paradigm claiming realities being social constructions. The researcher builds a theory as an outcome of an interpretation of the participant’s story. Accordingly, that meaning is a co-creation between the researcher and the participant [26].

### Recruitment procedures and participants

In 2021, those individuals ≥ 65 years old (*n* = 12) who had a diagnosis of advanced dry AMD in one or both eyes, causing a visual acuity of no more than 0.3 in decimals (6/18 or worse) in the best eye (0.8–1.0 as decimals corresponds to normal vision where 1.0 in decimals is 20/20), living in their own homes and with the cognitive ability to participate in the study and understand and communicate freely in Swedish were invited to participate in this study. Responsible low-vision therapists and other staff within the low-vision clinic in Halland County in Sweden were informed orally and in writing by the first author (JK) about the study’s purpose and structure. According to patient records at the low vision clinic, 66 individuals with advanced dry AMD were found, of whom 46 individuals met the above-mentioned inclusion criteria. The individuals were previously diagnosed by an ophthalmologist and referred to the low vision clinic since their visual acuity was 0.3 in decimals or worse. Recruitment and inquiries about participation occurred during a regular visit to the low-vision clinic or when a therapist contacted an individual by telephone and asked about participation. The low-vision therapists called 25 by phone, and 19 agreed to be contacted by the researchers. Of the participants in the study, eight were recruited when visiting the low-vision clinic and four by telephone. One participant was recruited via one of the researchers after this participant heard about the study and asked to participate. The sample included nine women and three men aged 65–87 with advanced dry AMD and a median age of 81 years. The informants were contacted by phone by the first author (JK) with an invitation to participate in the project including the individual interviews taking place before the intervention [24] and informed that participation is voluntary and can be interrupted without explanation and without affecting the participants’ continued care. Participants also received information about personal data management according to General Data Protection Regulation (GDPR), namely how the participants’ confidentiality would be ensured, how the data would be managed, and that no one outside the research group would have access to the data. Written informed consent was obtained from all informants before the individual interview was conducted. Reasons for not participating were hospitalisation, illness, and having to care for close relatives.

The Swedish Ethical Review Authority approved an ethics application for this study (EPN 2021/02877). All the ethical principles for medical investigation involving human beings in the Helsinki Declaration of the World Medical Association were followed.

### Manual

The first author conducted the individual interviews between August and September 2021 in the participants’ homes or wherever they preferred, such as a library or at the university. Data were generated with broad, open-ended questions and followed up with probing questions. The open-ended questions were designed to allow the participants to briefly describe their everyday life experiences living with a visual impairment caused by advanced dry AMD. The opening question was general; “*may I ask you first to tell me briefly what a typical ordinary day looks like for you?”* This was followed by furthermore specific questions, for example, *how AMD affects their daily life*, *what is particularly valuable to them*, *housework*, *their social life*, and *physical activity.*

Field notes were made during and immediately after each interview. The average length of the interviews was 37 min (R = 21 to 62 min), and all were digitally recorded with participants’ consent. After recording, the participants often relaxed, and more informal talk yielded valuable data. Repeated interaction between interview data production, analysis, and theoretical sampling was performed, also called an iterative research process [25].

### Data analysis

The analysis was completed in line with the process set out within constructivist grounded theory [23, 26]. All authors took part in the manual analysis together to ensure that the focus remained on the research direction, and discussion was held about coding, categorisation, theory building, and alternative interpretations. The manual data analysis began after the first interview and continued in an iterative process until the last interview. First, each interview was listened to several times by all authors separately to develop a sense of the overall context of the data and to gain familiarity with the detailed information provided. Together, we used our different disciplinary perspectives (nursing and sports science focusing on health promotion) as tentative tools for developing our ideas about the processes we defined in our data. Second, the focus shifted to initial coding, where we decontextualised our qualitative data into discrete excerpts and created codes to label them. In this phase, we also asked questions about the data, such as “What is happening?” and “What are the processes that take place?” Analytical reflective memos were written throughout the research process and used to note interpretations, conceptual connections, and patterns as they became evident to us throughout this process. Third, we made reflective notes (memoing) about ideas and concepts and their relationships; subcategories and properties were constructed by constantly questioning data and establishing the connection between what was discovered in the data. Fourth, focused coding was performed to explore preliminary subcategories in a comparative and iterative process. The tentative subcategories were created by constantly comparing for similarities, variations, and differences in data, codes, and subcategories, known as a constant comparative method [25]. Fifth, theoretical coding was used to deepen the analysis, produce a core concept and categories, and develop the theory. In this process, we tested new ideas to see how these concepts fit the collected data by constantly checking and rechecking. We also included the existing literature and theories to generate hypotheses that were then verified with existing empirical data to arrive at the most plausible explanations. All quotations were translated from Swedish into English and adjusted for readability. Data collection was continued up to the point of data saturation, which is fulfilled, when all possible interrelations among different aspects of the process are identified.

## Results

### The theory of Perpetuating ability to live life as usual

**Fig. 1:**
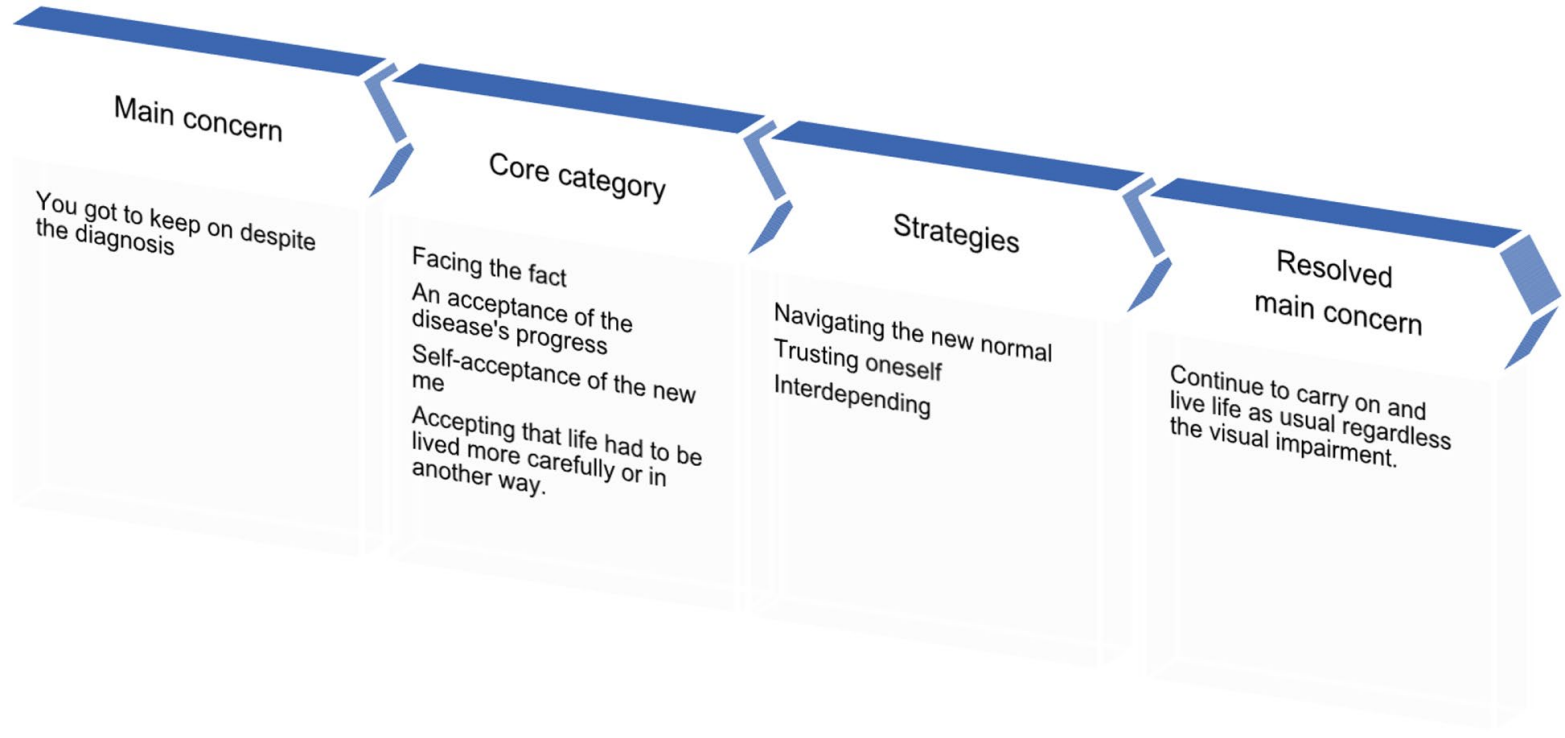
The theory of Perpetuating ability to live life as usual

The findings suggest that living with visual impairment caused by AMD was associated with challenging experiences in almost every aspect of the person’s everyday life (Fig. 1). The participant’s main concern was expressed as “You got to keep on” despite the diagnosis, and thereby, the core category was named “Facing the fact.” The core category elucidates that life cannot end just because of AMD; therefore, you must keep on trying to live as usual despite the progress of AMD. This involves a desire and perseverance to maintain everyday life, including the ability and opportunity to continue to live life as usual regardless of the visual impairment.

*“I want so much; I don’t want to quit” (no.10, male)*

The participants face an acceptance of the disease’s progress, self-acceptance of the new me, and the realisation that they must live their new life a little more carefully or in another way. The main concern was resolved using three strategies and processes: ^1).^ Navigating the new normal; ^2).^ Trusting own ability, and ^3).^ Interdepending. These strategies and processes involve replacing and simplifying, adjusting and adapting, getting support and being independent, developing courage, performing a task successfully, and being perseverant.

### Three subordinated categories of the core category that describe strategies used

#### Navigating the new normal

Facing vision loss meant the participants had to navigate the new normal and self-define themselves as the new me with a visual impairment. This meant finding themselves in a new situation by trying to *replace*, *simplify*, *adjust,* and *adapt* to the vision loss—both in achieving environmental mastery involving physical space and navigating their new social life.

There were no problems navigating in known physical areas, such as the home. The participants reported feeling safe at home as it was a familiar environment where they knew every corner.

*“You get your routines at home; I could do most things and with closed eyes. I do everything at home—I know every nook.” (no. 1, female)*

However, visual impairment greatly impacted day-to-day tasks, and the participants reported varying adjustment levels to their vision loss but also by simplifying. Some strategies reported included using magnifiers and flashlights to help with daily activities, such as reading the display on the washing machine or preparing food. Others used tactile stickers on their electric stove and washing machine, but when there was no resistance when turning the button and choosing the washing programme, it became difficult despite the stickers. To improve functioning in everyday life, they also chose to simplify activities.

*“I don’t make the same complicated dishes anymore; I take it a little easier.” (no. 7, female)*

Despite the various arrangements, sometimes things did go wrong, but often they took things as they came and with a laugh. Most of the participants took a walk in their nearby surroundings every day. However, even in familiar surroundings where they moved freely, both with and without walking aids, “the new normal” meant they had to navigate and live a little more carefully or in another way. AMD meant that they had difficulties perceiving contrasts, judging stances, and differences in height, which meant that they, even if it was a known area, required total concentration, continually looking down when they were walking outdoors.

*“When I’m out and walk, I’m fully concentrated on what I’m doing.” (no. 1, female)*

Accordingly, unknown areas were an even greater risk, as they had to be aware of hazards that significantly affected their mobility when walking, cycling, or driving. Some used a white cane when they were out walking in unknown areas. Using a white cane made others aware they were meeting someone with visual impairment. Otherwise, they felt the need to explain themselves and sometimes ended up in a defensive position. A couple of the participants cycled, and one wandered with her eyes to detect obstacles, as facing the deteriorating vision required adjustments to changing abilities and functional limitations. One of the first signs of AMD was that they had difficulties in dim light, and a prerequisite for navigating was having good light. Participants reported feeling fearful of falling, which made them stop going out in the evening or driving their car after dark. Vision loss reduces the ability to drive or drive for long distances, and some expressed the loss of driving as a loss of independence and sorrow.

*“It is a great sorrow not being able to drive a car; it limits life a little.” (no. 7, female)*

To perpetuate the ability to live as usual and avoid being prevented from doing things they wanted to, like traveling abroad, they chose to travel to familiar places they had visited several times before. They also adjusted and changed the way the activity was performed or replaced it. For example, to watch TV, they had to move closer and replace programmes that were not in Swedish because they could not read the subtitles. Some of them tried listening to the audio description for blind people on the TV, but they all thought the detailed explanations made them confused, making them unable to follow the programme in real-time. All participants mentioned how their vision loss reduced their reading ability and reported using other senses to compensate for their vision loss. It was common to listen to books or the radio or have a relative read the morning paper and inform them if anything had happened.

*“I have read (books) a lot in my life, but now I listen, and then I listen to the newspaper.” (no. 3, female)*

Most participants also reported that the vision loss associated with AMD led to a loss of ability to engage in hobbies and leisure activities such as sewing, fishing, skiing, tennis, or activities with friends. After adjusting for a while, they could continue with their activities, but as the disease progressed, it got worse, and finally, they had to stop.

*“I used to play a lot of tennis and downhill skiing – I cannot do anything now.” (no. 6, male)*

Some participants adapted to their new condition to continue with their usual activities even though it had to be in a completely different way. For example, a woman who liked reading and participated in a book circle stated that she continued doing so, but in a different way, which this quote illustrates:

*“I still participate in my book circle. I can’t read, but I listen and drink coffee.” (no. 5, female)*

Some of the participants were confronted with strange visual hallucinations, which terrified them and prompted them to visit the emergency unit. Cartoon characters, a jungle with flowers and greenery, or visual effects of green and pink lines and quadrants appeared and caused fearsome feelings when participants were unsure of what caused them. Even though hallucinations are rather common among older adults, whose low vision is caused by age-related macular degeneration, the staff at the emergency unit at the hospital did not know what it could be. One woman was recommended to get an x-ray, another was told that they did not know what was wrong, while a third met an ophthalmologist who knew what it was. Since the hallucinations were frightening, they thought it would be appropriate to acquire information about how hallucinations caused by the brain’s adaptation to significant vision loss in AMD can occur, as this allowed them to navigate the new normal without feeling terrified.

#### Trusting own ability

It was deemed necessary for the participants to trust in their abilities to deal with various situations. Trusting own ability includes *developing courage* since the deterioration of existing vision could be frightening, embarrassing, and lead to difficulties. The participants reported that their vision made it difficult to distinguish between and recognise faces and other people, leaving them not knowing whom they had just met, causing embarrassment and worry about appearing to be snobbish. The visual impairment also made it difficult to catch up with others when travelling as they had to be careful and not fall. Sometimes, this made them lose confidence in social situations and therefore withdraw from social activities they appreciated.

*“If you meet acquaintances, you don’t recognise them. Many times, it is annoying when they think that you don’t want to say hello, I find that difficult.” (no. 7, female)*

Moreover, trusting own ability the category also meant that the participants had to trust their ability to perform specific tasks, carry on, and live their lives as usual regardless of the visual impairment. This involved daily routines like getting up in the morning, making the bed, emptying the dishwasher, cleaning, and walking around the block, as one of the participants explained:

*“It is important to fend for myself, cook, wash myself and clean, yes simply fend for myself.” (no. 5, female)*

They also reported actively working to counter the functional limitations caused by their visual impairment. To maintain confidence and trust in their ability, some challenged themselves not to stop doing what they have always managed. However, it was challenging; it was a kind of *perseverance* not to give up their life as it used to be before their visual impairment.

*“I try to cook; I’ve been doing that all my life. Sometimes things go wrong. Last Saturday I baked bread, and I was so proud of that. At least I managed to make a loaf of bread and I’m happy about that.” (no 3, female)*

The quote above illustrates how everyday life may change through increased abilities that contribute to trust in oneself through a sense of achievement and how this can significantly improve a person’s perceived autonomy. The most effective way of developing a strong sense of trust in one’s own ability was by *performing a task successfully*. Most participants attempted to take on challenges without becoming overwhelmed by negative emotions and maintain their everyday tasks, engaging in life, despite the visual impairment. When something went wrong, they often took it with humour, which made it easier to cope, such as when one participant with a laugh told us about taking sprinkles instead of spices when cooking. Continuing to do things as they did before comprising a *perseverance* not to give up. One woman was engaged in voluntary activity, which increased her mastery and added meaning to life. However, she had to adapt to the deterioration of vision and stop working in the cash register, choosing instead to unpack the donated goods. Nevertheless, she kept on going with her voluntary work. One participant reported a refusal to listen to audiobooks. This participant was determined to keep reading books (only about ten lines at a time) despite significant difficulties, even when using a magnifier. However, the impact of vision loss did not stop the participants from getting out into their community.

#### Interdepending

A significant concept was interdepending. The concept of *interdepending* refers to an interplay and cycling process between receiving support and *being independent*. To be able to keep on, as usual, being independent and managing one’s everyday life was a stated desire. However, in this study, being independent requires a team effort that entails *getting support* from others, which created a sense of interdependence and closeness with family and friends. One woman described her daughter’s support “as if she was her eyes”.

The findings show that, besides family members, friends were important and valuable support. Helping with daily activities and errands, providing transport and informal company, and reading different instructions and messages represented substantial support. Receiving support from others reduced the impact that AMD had on their daily lives. This was especially true for those who did not live with a partner and lived alone, making them more vulnerable.

For example, for those participants who lived alone, there was a fear of being unable to keep everything clean or that their home would be perceived as dirty by people visiting them. Their visual impairment made them worried about hygiene, that is, being unable to see dirt. One of the women illustrates this viewpoint:

*“I think that I haven’t spilled on myself today. I usually tell my sister to tell me if it’s too dirty at home.” (no. 12, female)*

Living on their own required that they had someone to contact, and with whom they had staying in touch by telephone frequently. However, they did not want to take up too much time or bother others, especially if it did not involve close relatives. For those who lived with a partner, the partner meant everything. They were two who lived together for many years, and the partner completed what the other person had difficulties with because of impaired vision. Likewise, the partner made it possible to manage and remain living in their house. One of the men said:

*“It’s valuable that there are two of us; I wouldn’t be able to cope without my wife because I can’t see—it hadn’t worked.” (no. 2, male)*

The findings furthermore show that there was a loss when the partner passed away, not only regarding the longing for the partner but also the support they received in their everyday life due to impaired vision.

The participants lived quite active lives, but desired support from others, especially in the terms of transport. Transporting service and being picked up and dropped off outside the home was a prerequisite for those living alone to be able to participate in society and social activities. However, sometimes the drivers at the taxi service for the disabled did not understand what visual impairment meant and what challenges the older adults are confronted with had to face. Therefore, in some cases they were dropped off and had to manage on their own to get where they were supposed to go.

## Discussion

Our study explored the lived experiences of a small group of older people with advanced dry AMD. The data were rich and, in many ways, consistent. For that reason, it was possible to generate the theory of *Perpetuating ability to live life as usual* by grounding it in data (Fig. 1). The theory might be transferable to both older people with advanced dry AMD and those with severe visual impairment.

The study’s findings demonstrate that living with AMD could be challenging, but simultaneously, the participants expressed that life cannot end just because of AMD. Instead, they needed to face the facts and undertake various strategies to carry on and live life as usual regardless of their visual impairment. According to [26], this could be described as a way of accepting a new life situation and maintaining or increasing quality of life.

When their vision deteriorated, daily life was affected in various ways. The obvious things they could do earlier, such as reading and driving cars, needed to be re-evaluated, although they could still manage daily activities. However, they needed to use different strategies to adapt and adjust by doing things in a new way in order to preserve life as usual, such as cooking less complicated dishes, staying in familiar areas when going out, or using magnifiers when reading. When magnifiers no longer worked, they listened to audiobooks or radio. This is in line with another study [27], where older visually impaired adults found new ways of solving familiar things by both adjusting and adapting.

Navigating the new normal can be interpreted as successful problem-based coping [28], which is helpful when dealing with new life situations. Navigating helped the participants preserve everyday activities, which is essential for maintaining independence and autonomy and increasing well-being [28]. Moreover, performing a task successfully increases a person´s self-efficacy [29], and preserving independence despite visual impairment is acknowledged as crucial [29]. However, AMD does not appear suddenly; it is a progressive disease [6]. Even though the impairment worsened, our participants´ preserved their independence by using support as a kind of interdependency, although receiving support may imply a vulnerability and a fear of how others perceive them [30, 31]. This can lead to not telling others about their impaired vision [27]. Another reason remaining silent is to maintain control of one´s life without being affected by others’ concern or benevolence [32] or to avoid burdening family or friends [27].

Despite difficulties, the participants in our study persevered and continued to participate in activities at home and places outside their homes, even if they were dependent on others by *trusting their own ability*. This is essential, because continuing with activities and social relationships strengthens older adults’ self-confidence and independence despite the obstacles caused by visual impairment [32, 33]. Based on the older adults’ beliefs society can set up meeting arrangements, such as making it easier for older people to participate in organised activities.

According to Hill [34], an optimistic way of thinking regarding the decline of the aging body involves mobilising an individual’s unknown power and flexible behaviour. This also supports well-being by, for example, dealing with hidden or undetected difficulties. An optimistic outlook, independence, activity, and adjustment are essential to life satisfaction, as well as aging in a successful way [35]. Nevertheless, a balance between expectations, aspirations and their perception of life is crucial concerning overall satisfaction with life, especially among women where successful aging is enabled by having a positive perception and perspective, when having to deal with unforeseeable challenges difficulties [36].

Despite strategies such as *replacing*, *simplifying*, *adjusting,* and *adapting* to vision loss, the participants in our study reported feeling vulnerable. Some of the participants in our study had experienced different kinds of hallucinations, which at first made them afraid conveyed a feeling of being crazy despite their normal usual mental state. The experience was probably caused by Charles Bonnet syndrome (CBS), which is relatively common among patients with AMD, and there are likely hidden statistics [37]. However, CBS is not always known to physicians [38], and in our study, the hospital staff at the emergency unit did not recognise the symptoms. This shows the importance of informing patients, especially those with AMD, of such visual phenomena and what causes it to prevent feelings such as fear. The participants also expressed a fear of falling, which made them stop performing some activities, increasing the risk of functional decline, falls and injuries, and mortality [18–21].

### Limitations of the study

Our study has potential limitations. The population was a small group of 12 older adults recruited from a single low vision clinic which may have an impact on the results. Moreover, education levels, income, and presence of other chronic diseases than AMD was not considered which may have influenced the data from the interviews. Even though the results were consistent, it would be beneficial whether there was a more even distribution between men and women. The men in our study were all more than 81 years old which also may have an impact. Despite the mentioned potential limitations, we were able to capture various perspectives of daily life among older adults with advanced dry AMD. However, as a part of the project, an intervention of empowerment-based physical activity intervention for people with advanced dry AMD follows where even more data are collected and reported.

## Conclusion

This study offers unique insights into what it means for older adults to live with advanced dry AMD, which affects their central vision, causing difficulties in daily life activities such as reading, performing detailed tasks, recognising faces, and driving. We discovered that the participants’ main concern was “you got to keep on” under the impact vision loss. The theory of *Perpetuating ability to live life as usual*, explains how they resolved themselves to “*facing the fact*,” involving a desire and perseverance to carry on and live life as usual since life continues despite the visual impairment. This grounded theory offers a tool to understand how the participants used the following strategies and processes. *Navigating the new normal* involved *replacing and simplifying*, and *adjusting and adapting*, and *trusting own ability* involved *getting support and being independent*, while *interdepending* meant *developing courage, performing a task successfully* and *being persistent*.

The results from this study have the potential to capture unique and valuable knowledge about older people’s responses and behaviour concerning vision-related issues caused by AMD, which accumulates and consequently also has an impact on their quality of life. Such a study also gives rise to implications for research and practice in strengthening older people with AMD meeting future challenges.

## Data Availability

- All data generated or analysed during this study are included in this published article - The datasets generated and/or analysed during the current study are not publicly available to preserve individuals’ privacy according to the Swedish law *Act (2003:460) on ethical review of research involving humans* (http://www.eurecnet.org/information/sweden.html) but are available from the corresponding author on - All data was coded so that only relevant researchers can access it. Data are locked in a safe at the university (School of health and welfare, Halmstad University), away from the code key, which is locked in another safe. Research material is saved for ten years after the research principal completes the project. Only researchers who belong to the project have access to the raw data. All information that can reveal the identity of the participants are pseudonymised.
